# Varicocele

**Published:** 1883-04-20

**Authors:** A. J. Howe


### VARICOCELE.

By A. J. Howe, M. D.

So variable are the opinions at present prevailing in regard to the treatment of varicocele, that I am induced to reiterate what I have said in other places, and to add what a more extended experience has thrown in my way. In a late issue of the Cincinnati Lancet and Clinic, Dr. Dawson, a surgeon of excellent attainments, urges the necessity of excision (castration), and denounces more conservative means. Others of equal eminence advise ligation of the tortuous veins, either openly or subcutaneously. Ingenious are the methods devised to strangulate the varices, yet all are attended with some risk through phlebitis and abscess.

I will remark incidentally that not one case of varicocele in a hundred needs other treatment than an efficient suspensory bandage. In some instances the use of astringent washes contracts the scrotal integument, and thus results some benefit. It is a professional mistake to advise severe and dangerous treatment for a defect that is scarcely inconvenient.

A slight degree of varicocele exists in every man. The venous dilatation comes from a disadvantageous vascular arrangement on the left side of the genital and urinary apparatus. The left emulgent vein is obstructed by the pressure of the abdominal aorta; and the spermatic vein is not valved at its confluence with the emulgent.

Sometimes the varicose state is seen in the left leg, in the scrotal integument, and in the skin of the abdomen. The left iliac vein is not as well emptied as its associate on the opposite side. Young men, finding the spermatic cord enlarged, or its walls distorted with coils of tortuous and dilated veins, are apt to suppose that a serious lesion exists, and to think that an advertising doctor is the most likely to save them from impotence and degradation.

How much unchaste practices may have to do with varicocele I am unable to say. However, I think they have less influence over the origin and aggravation of the disease than is generally supposed. It is senseless to accuse a young man of illicit practices because a pronounced degree of varicocele is present. The most prudent of men may have troublesome varices of the spermatic cord.

It has been observed that those males who possess pendulous scrotal pouches are the oftenest afflicted with varicocele; and that the affected testis has a long cord, and hangs correspondingly low, the organ resting on its side in the bottom of the scrotum. In not a few instances the epididymis is covered with varices, and the testicle seems diminished in size. In such cases the individual suffers from shooting pains in the cord, and dragging sensations in the testicle and inguinal region of the side affected.

As has been intimated, an efficient suspensory apparatus, which costs a dollar or two, and is for sale by all apothecaries, will afford the relief needed in the majority of cases. But aggravated forms of the disease present themselves. Such are to be operated upon if the possessor demands a radical cure.

There is a moiety of danger in the operation, yet not enough to deter the surgeon from entering confidently upon its execution. The patient should be anæsthetized to a degree of insensibility, and then the operator seizes the disordered testicle and cord in his left hand, and his right, holding a scalpel, make a free incision along the course of the cord, the cut beginning an inch below the spine of the pubis and ending near the epididymis, the knife dividing the dartos. The dilated and tortuous veins bulge into the wound, where they may be excised with sharp shears or large scissors. As soon as a plexus of varices is removed, the veins of the cord are scarcely seen. The blood flows out of them, and their walls collapse. The next step is to excise a liberal amount of scrotum on that side. The fault is not in taking too much, but in removing too little. After all cutting is over, the borders of the scrotal wound are to be joined by the use of a glover's needle, and a fine silver wire utilized as a thread in common sewing. The over-and-over stitch is the one to be employed. At the end of two weeks the loops of the wire may be clipped with scissors, and removed in segments. The wound is to be dressed daily with "boro-glyceride with thymol." The suppuration will be moderate, and the pain readily endurable. No hemorrhage of a startling nature need be apprehended. Sometimes in operating the testicle gets liberated from its vaginal tunic, and tumbles about freely. Such a complication should be avoided, yet I have not known the accident to result in harm.

After the healing process is complete, the testis hangs high, and does not drag on the cord. In fact the traumatic surfaces of the cord and scrotum rest in contact, and join during the reparative activities. A suspensory may be employed for a few weeks or months, yet it is not necessary.

I have operated in the way described dozens of times, and in no instance with bad results. The procedure is not as dangerous as castration, and the functions of the testicle are fully retained.

I may remark that the hair in the vicinity of incisions should be snipped off to avoid their getting into the wound, and by their presence preventing union by the first intention.

I should say that I do not remove the varices so completely or thoroughly as in former operations. I have found that the excision of a few loops will do, especially if a generous supply of scrotum be taken away.—*Eclectic Med. Jour.*

---

A BOTTLE of fifty gallons' capacity, the largest ever blown in this country, was lately made at Millville, N. J.

